# Spaceflight-induced neuroplasticity in humans as measured by MRI: what do we know so far?

**DOI:** 10.1038/s41526-016-0010-8

**Published:** 2017-01-10

**Authors:** Angelique Van Ombergen, Steven Laureys, Stefan Sunaert, Elena Tomilovskaya, Paul M. Parizel, Floris L. Wuyts

**Affiliations:** 1 Antwerp University Research Centre for Equilibrium and Aerospace (AUREA), University of Antwerp, Groenenborgerlaan 171, Antwerp, 2020 Belgium; 2 Faculty of Medicine and Health Sciences, University of Antwerp, Universiteitsplein 1, Wilrijk (Antwerp), 2610 Belgium; 3 Faculty of Sciences, Department of Biomedical Physics, University of Antwerp, Groenenborgerlaan 171, Antwerp, 2020 Belgium; 4 Coma Science Group, GIGA-Research & Neurology Department, University and University Hospital of Liège, Liège, Belgium; 5 KU Leuven—University of Leuven, Department of Imaging & Pathology, Translational MRI, Leuven, Belgium; 6 SSC RF—Institute of Biomedical Problems, Russian Academy of Sciences, Moscow, Russia; 7 Department of Radiology, Antwerp University Hospital & University of Antwerp, Antwerp, Belgium

## Abstract

Space travel poses an enormous challenge on the human body; microgravity, ionizing radiation, absence of circadian rhythm, confinement and isolation are just some of the features associated with it. Obviously, all of the latter can have an impact on human physiology and even induce detrimental changes. Some organ systems have been studied thoroughly under space conditions, however, not much is known on the functional and morphological effects of spaceflight on the human central nervous system. Previous studies have already shown that central nervous system changes occur during and after spaceflight in the form of neurovestibular problems, alterations in cognitive function and sensory perception, cephalic fluid shifts and psychological disturbances. However, little is known about the underlying neural substrates. In this review, we discuss the current limited knowledge on neuroplastic changes in the human central nervous system associated with spaceflight (actual or simulated) as measured by magnetic resonance imaging-based techniques. Furthermore, we discuss these findings as well as their future perspectives, since this can encourage future research into this delicate and intriguing aspect of spaceflight. Currently, the literature suffers from heterogeneous experimental set-ups and therefore, the lack of comparability of findings among studies. However, the cerebellum, cortical sensorimotor and somatosensory areas and vestibular-related pathways seem to be involved across different studies, suggesting that these brain regions are most affected by (simulated) spaceflight. Extending this knowledge is crucial, especially with the eye on long-duration interplanetary missions (e.g. Mars) and space tourism.

## Introduction

More than 50 years of manned spaceflight have taught us that space is a hostile environment for human health; microgravity, ionizing radiation, absence of circadian rhythm, confinement and isolation are just some of the stressors space travelers encounter.^1,2^ Obviously, all of the latter can have an impact on human physiology and lead to detrimental changes.^3^ An example of this is the microgravity-induced cephalic fluid shift, which has been thought to cause to a wide range of symptoms such as increased intracranial pressure, visual impairment (named the visual impairment intracranial pressure syndrome, VIIP syndrome)^4,5^ and alterations in cerebral oxygenation^6^ and cerebral blood flow (CBF)^7,8^ (for a full synthesis on spaceflight-induced cephalic fluid shift, readers are referred to.^9^

It is important to acquire insight into the precise effect of spaceflight as this can aid in the development of adequate countermeasures and guarantee safety and efficiency in future space missions. Some organ systems have been studied thoroughly under space conditions, such as the cardiovascular,^10^ immune^11,12^ and musculoskeletal systems.^13,14^ Although there is an increasing interest on the effect of spaceflight on the human central nervous system (CNS),^15,16^ up to date, not much is known about the functional and morphological effects of microgravity on the human CNS. Previous studies have already shown that CNS changes occur during and after spaceflight in the form of neurovestibular problems,^17,18^ alterations in cognitive function and sensory perception,^19^ problems with motor function,^20^ cephalic fluid shift^9^ and psychological disturbances.^21^ For example, neurovestibular problems originate partially at the level of the peripheral vestibular organ that suddenly is deprived of the sense of gravity,^22–24^ so an intravestibular conflict emerges between the different angular and linear acceleration detectors. Therefore, one could hypothesize that this may also have an effect on the vestibular nuclei in the brain as well as on the cortical projections where sensory integration takes place between ‘disturbing’ vision, ‘altered’ proprioception and ‘conflicting’ vestibular information, such as the insular cortex, the temporo-parietal junction and the thalamus.^25,26^ In addition, it is known that the primary somatosensory and the somatosensory association cortical networks are involved in proprioception.^27^ Zero-gravity induced modifications in these network interactions could therefore underlie the deficits in sensory perception as seen in astronauts and vice versa.^28^ Also, the cerebellum is known to be involved in fine motor control, coordination and equilibrium^29^ and changes in cerebellar function and connectivity could therefore explain typically-seen motor coordination and movement-timing problems during and after spaceflight.^28^

In general, literature on the impact of spaceflight on space travelers has mainly focused on the extra-cerebral or peripheral systems, e.g. the musculoskeletal and the cardiovascular system. Yet, studies on CNS dysfunction are scarce. However, in the past few years more and more interest has been attributed to this topic. The latter is probably due to recent advances in structural and functional neuroimaging techniques over the past 20 years leading to a growing role of these technologies in Earth-bound medicine. Additionally, the increasing interest in interplanetary missions adds to the importance to probe the changes occurring in the human brain in relation to short- and long-duration spaceflight.

### Aim of this review

Previous reviews on spaceflight-induced neuroplasticity,^30–34^ dating from the 1990’s or early 2000, are largely based on animal studies and do not include more recent findings from more advanced neuroimaging techniques. Furthermore, the effect of space analogs -in particular head-down bed rest (HDBR)- on the human brain has received increasing interest in the past few years and has resulted in novel findings, some of which are translatable to long-duration spaceflight. An updated overview of this emerging literature could help to synthesize our current understanding, as well as to address the current shortcomings in order to direct and enhance future research.

### Neuroplasticity and how to measure it

Neural plasticity or neuroplasticity can be defined as the capability of the brain to alter its structure or function in response to exposure to new stimuli or environments. It is a crucial underlying component of skill learning in healthy individuals (i.e. learning-dependent or experience-dependent neuroplasticity) and functional recovery after injury.^35^ Neural plasticity can take place at several levels: from synaptic plasticity at the (sub)cellular level to plasticity at the system and network level.^35^ In this review, we will focus on systems plasticity across neural networks in human beings. Brain plasticity of the CNS can be studied with a number of methods. Examples of techniques commonly used in neuroplasticity studies are electroencephalography (EEG)/evoked potentials (ERPs), structural and functional magnetic resonance imaging (MRI) and transcranial magnetic stimulation (TMS). These techniques can be used to study the cortical dynamics, e.g. magnitude of task-related or resting-state neural activity, changes in activity patterns, representational map size and cortical excitability. Other commonly used techniques include positron emission tomography (PET) and magnetic resonance spectroscopy, but up until now, no space-related studies have been carried out with the latter techniques, so we will only describe the applied techniques related to real and simulated spaceflight.

When it comes to spaceflight, EEG is the most commonly used technique. This is associated with the portability of EEG and the fact that this technique can easily be used in extreme environments.^36^ In EEG, electrical activity of the brain is monitored and measured by placing multiple electrodes along the scalp. Examples of the use of EEG in regards to spaceflight are the studies on electrocortical activity in astronauts during spaceflight^37^ or in subjects during parabolic flights.^38^ EEG has a high temporal resolution, but on the contrary, it has a low spatial resolution making it tricky to attribute EEG findings to a precise cortical or subcortical region. Current state of the art neuroimaging techniques such as MRI, as further described below, have a high spatial sensitivity and therefore allow a detailed assessment of brain structure and function.^39^ We will not go into detail on EEG-based space studies on neuroplasticity, but we will focus on spaceflight-induced neuroplasticity as measured by MRI. However, EEG studies have been proven to be very useful in better understanding the effect of spaceflight and microgravity on the human brain and ideally, would be combined with functional MRI in a multimodal fashion to cover both temporal and spatial aspects of neuroplasticity as good as possible. Readers are referred to Marušič *et al*. for a recent and thorough review on EEG-based neuroplasticity studies in relation to spaceflight, microgravity and hypergravity.^36^

MRI is an imaging technique that allows measuring structural, functional, metabolic and vascular events in vivo. An example of an anatomical MRI-technique is volumetric T1-weighted anatomical imaging to assess regional differences of a specific brain region, i.e. gray matter (GM), WM and cerebrospinal fluid (CSF), between groups. A common technique to perform this type of brain morphometry is called ‘voxel-based morphometry’.^40^ Another MRI technique is diffusion tensor imaging (DTI). DTI is based on the molecular Brownian motion (i.e. diffusion) of the water molecules in the brain.^41,42^ Several local microstructures such as myelin, cell membranes and other organelles will limit free diffusion in the brain. The DTI MRI technique uses this limitation of free diffusion by measuring the diffusion path of water molecules. In DTI, it is assumed that the signal in each voxel can be described as a diffusion tensor. This diffusion tensor will determine the orientation of the longest axis of the ellipsoid, which will be ideally aligned with the orientation of the underlying white matter architecture. From the diffusion tensor, several parameters can be defined, such as fractional anisotropy (FA) and mean diffusivity. Therefore, DTI allows, up to some extent, to study the underlying white matter (WM) structure and microstructural features.^41,42^ A semi-automated procedure can now be implemented to connect neighboring voxels where the diffusion tensor points towards each other, and by doing so the underlying WM bundle can be reconstructed. This process is called diffusion tensor tractography.^43,44^ Another technique called tract-based spatial statistics, an automated and observer-independent approach, allows to assess FA in the major WM tracts on a voxel-wise basis across groups of subjects.^45^

Functional MRI (fMRI) is also a MRI-based technique in which stimulus or activity-induced brain patterns can be investigated. fMRI is based upon the fact that neural activation is associated with a local vascular response, constituting the blood-oxygen-level dependent (BOLD) signal. The magnitude of the BOLD-signal resembles the hemodynamic response and can indirectly be linked to the magnitude of neural activation in specific brain areas. fMRI has been crucial in the determination of functional organization in the human brain.^46,47^ A derivative of fMRI is the resting-state fMRI technique (rsfMRI) in which neural activity at rest, without any stimulus or activity, is measured. For a complete summary on the use of MRI-based techniques in neuroplasticity studies, readers are referred to.^48^

Lastly, TMS is a technique that allows stimulation of an area of the cortex non-invasively through the scalp by means of brief pulses, administered by a stimulation coil using time-varying magnetic fields.^49^ By doing so, alterations in cortical excitability can be induced and measured. For example, when TMS is applied over the primary motor cortex (M1), TMS can depolarize the corticospinal tracts and evoke contralateral muscle contractions.^49^ For a review on the use of TMS in neuroplasticity studies, readers are referred to e.g.^49^ or.^50^ TMS has been used previously to investigate corticospinal excitability in relation to hypergravity and microgravity, however, this was a preliminary investigation and data from only 3 parabolic flyers were included.^51^

### Ground-based space alternatives for human studies

Research on humans in space is complicated, expensive and subject to several logistic and payload restrictions. In addition, only few subjects can be investigated at the same time, leading to reduced study power and limited generalization. Therefore, space researchers have developed Earth-based models in which some aspects of spaceflight can be simulated in order to set-up investigations on a bigger scale and by which the difficulties of actual space research can be overcome.

Immersion was the first ground-based model ever used for investigating the consequences of spending time in a reduced gravity environment. Dry immersion involves immersing the subject in thermo-neutral water while being covered in an elastic waterproof fabric in order to keep the subject dry and to overcome the unpleasant consequences of long-term direct water exposure.^52^ Immersion is an adequate spaceflight alternative, since it mimics several spaceflight features such as ‘supportlessness’ (i.e. lack of a supporting structure against the body), centralization of bodily fluids, confinement, immobilization and hypokinesia.^52^ Although dry immersion is a good model, it is not (yet) widely implemented and so far, it has not been used to quantify the neural changes associated with it. For a more general review on dry immersion and its implementation, readers are referred to.^52^

HDBR is an acceptable, reliable and the most implemented alternative to simulate most of the changes occurring due to spaceflight, both of a physiological^53,54^ and a psychological kind.^55^ In principle, HDBR consist of a subject being in a bed that is inclined with the head down (−6° in most cases). This can be done for short-term investigations (e.g. 72 hours in^56^) or long-term studies (e.g. 90 days in^57^). The head down tilt induces an upward fluid shift, similar to the one seen in space. Spaceflight-induced cephalic fluid shift is thought to cause a wide range of symptoms such as increased intracranial pressure, visual impairment (together named the VIIP syndrome), alterations in cerebral oxygenation and changes in CBF. In addition, HDBR is also characterized by immobilization, inactivity and confinement. This leads to equivalent alterations as seen in spaceflight in calcium homeostasis, musculoskeletal deterioration (e.g. muscle loss and changes in bone architecture) and a psychological load, respectively. For a review on bed rest and its application in space research, readers are referred to the publication by Pavy-Le Traon and colleagues.^54^

**Fig. 1:**
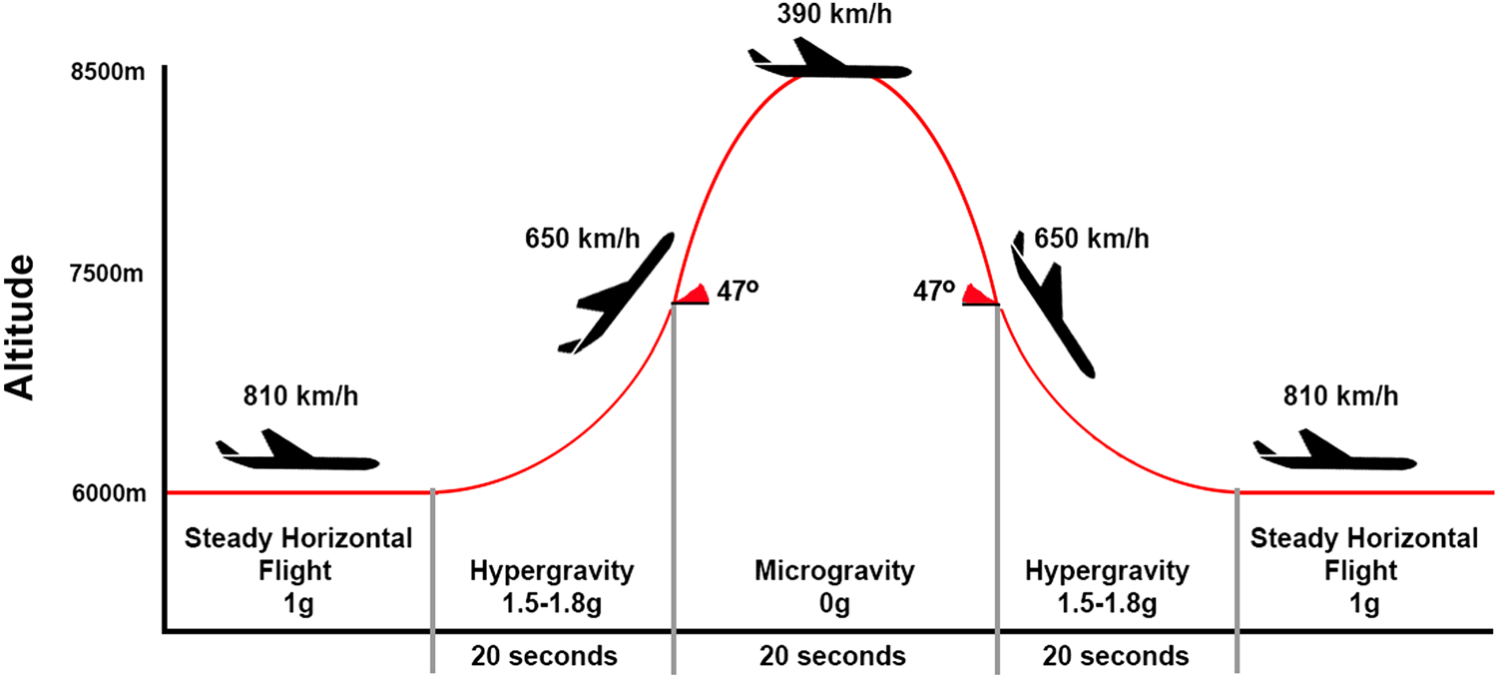
Typical flight trajectory of a PF for 0 g parabola’s

A third “ground-based” alternative to spaceflight is parabolic flight (PF). During a PF, a specific flight trajectory wherein the acceleration of the aircraft cancels the acceleration due to gravity is carried out. By doing so, normo-, hyper- and microgravity phases are alternatingly experienced by the subjects on board of the PF aircraft. The hypergravity phase precedes and follows the microgravity phase and is characterized by 1.5 to 1.8 g and lasts around 30–35 s. The microgravity phase on the contrary resembles 0 g during which approximately 0 g is experienced lasting around 20–25 s (Fig. 1). In addition, the flight profile can be modified to fly parabolas of Martian gravity (0.38 g) and lunar gravity (0.16 g). In between parabolas, the aircraft flies in normal 1 g conditions. In general, one PF consists of 31 parabolas and lasts around 3 to 3.5 h. For more information on the underlying dynamics of a PF, readers are referred to the paper of.^58^ Important to note is that PF is the only Earth-based method that allows researchers to conduct life science studies in microgravity.

Another approach to mimic spaceflight-related features, is to investigate human deployment analogs, such as Antarctic overwintering, undersea missions, etc… Sensory deprivation, high stress loads, confinement, isolation and shifted circadian rhythm are all replicated to high fidelity and therefore, these missions form an acceptable spaceflight analog (except for space-related changes in gravity). Furthermore, space mission simulation studies in the form of isolation missions, e.g. the MARS500 study, can also be used as a spaceflight analog, in particular to investigate the effects of long-term isolation and confinement. An example hereof is the assessment of peripheral and central (assessed by means of EEG) stress markers in the MARS500 mission.^59^

### Search method

For this review, the Medline (PubMed) and EMBASE databases were searched for papers using the term “spaceflight”, “microgravity”, “bed rest”, “PF”, “dry immersion” or “head-down tilt” and “brain”, “neuroplasticity”, “neuro”, “MRI”, “DTI” or “fMRI” without restriction of publication date. Reference lists from retrieved articles were also searched manually for relevant publications that were not included in the lists created through the Medline database. Non-English studies were excluded. The abstracts of the resulting articles were screened to select the relevant articles, i.e. articles describing new findings on spaceflight-induced neuroplasticity or commenting on previously reported results in the field. Only studies on human subjects were included. As stated above, EEG-based studies were excluded from this review.

## Overview and critical appraisal of the current literature

**Fig. 2:**
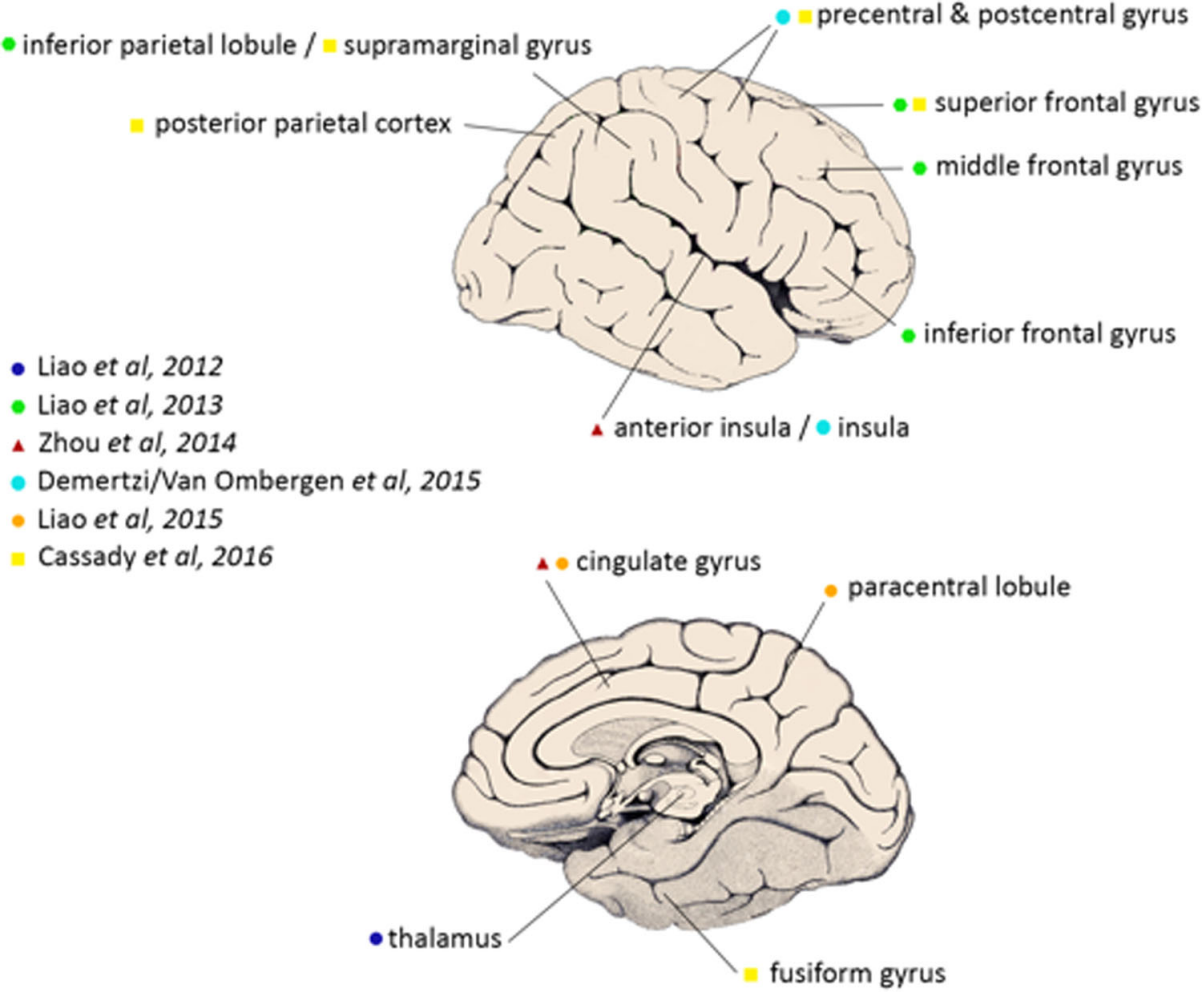
Cortical and subcortical brain areas most affected by spaceflight analogs or actual spaceflight, as described in the rsfMRI studies discussed in this review (figure modified after^26^, with permission, originally from^111^). For simplification, laterality of the findings was not taken into account. A more extensive description of the findings can be found in Table 1

A synthesis and critical appraisal of the MRI-based studies included can be found in Table 1. For clarification, a summary of brain regions found to be affected in (simulated) microgravity can be found in Fig. 2.

**Table 1:** Synthesis and critical appraisal of the existing literature on spaceflight-induced neuroplasticity

Study	*n*	Protocol	Main finding(s)	Limitation(s)
Roberts *et al.* ^57^	4	fMRI (1.5 T) and TMS before and after 90 days of −6° HDBR.	- Decrease in fMRI signal during motor activity in the leg (non-significant).- Decrease in corticospinal excitability measured by TMS immediately after HDBR.- Return to baseline corticospinal excitability measured by TMS 2 weeks after HDBR.- Relation between corticospinal excitability and functional mobility.- Inter-subject variability.	- Small sample size; no generalization possible.- Relatively young age in comparison to astronauts.- No control group.
Liao *et al.* ^56^	12	rsfMRI (3 T) before and after 72 h of −6° HDBR.	- Decreased ALFF in left thalamus (including medial dorsal nucleus and ventral lateral nucleus).	- Short-term.- Relatively young age in comparison to astronauts.- No correlation with behavioral tasks investigated.- No control group.
Liao *et al.* ^61^	12	rsfMRI (3 T) and mental transformation tests before and after 72 h of −6° HDBR.	- Decreased ReHo in R IFG, L IPL in HDBR.- Increased ReHo in BL MFG and L SFG in HDBR.- Correlation between mean ReHo in L IPL and mental transformation task in HDBR.	- Short-term microgravity.- Relatively young age in comparison to astronauts.- No control group.
Rao *et al.* ^63^	16	fMRI (3 T) and BART data before and after 45-days of −6° HDBR.	- No significant changes in risk-taking behavior.- Less deactivation in VMPFC after HDBR than before.	- Relatively young age in comparison to astronauts.- No control group.- HDBR-related stress might influence risk-taking behavior.
Zhou *et al.* ^65^	16	rsfMRI (3 T) before and after 45 days of −6° HDBR.	- Decreased DC in L aINS and MCC.- Decreased positive RSFC between L aINS and MCC and SMA, between L aINS and frontal cortex after HDT.- Decreased positive RSFC between MCC and L insula, R insula, R IFG, R lateral SFG, R precentral gyrus.- Decreased negative RSFC between MCC and medial SFG.	- Relatively young age in comparison to astronauts.- No correlation with behavioral tasks investigated.- No control group.
Demertzi/Van Ombergen *et al.* ^60^	1	rsfMRI (3 T) before and after 169 days of spaceflight.	- Decreased RSFC in R insula.- Decreased RSFC between L cerebellum and R motor cortex.	- Single case; no generalization possible.- No correlation with behavioral tasks investigated.
Liao *et al.* ^66^	20	rsfMRI before, during and after 7 days of −6° HDBR.	- Decreased ALFF in PCC on HDBR1 and HDBR5 and in L paracentral lobule on HDBR2, HDBR3 and HDBR7 compared to during HDBR.- Increased ALFF in ACC on HDBR2, HDBR4, HDBR5 and HDBR7 and in L cerebellum posterior lobe on HDBR3 and HDBR7 compared to during HDBR.	- Short-term.- Relatively young age in comparison to astronauts.- No correlation with behavioral tasks investigated.- No control group.
Li *et al.* ^68^	18	MRI (3 T, VBM and TBSS) before and after 30 days of −6° HDBR.	- Decreased GMV in BL frontal lobes, temporal poles, parahippocampal gyrus, insula and R hippocampus.- Increased GMV in vermis, BL paracentral lobule, R precuneus, L precentral gyrus and L postcentral gyrus.- No changes in WM after correction for multiple comparison.	- Relatively young age in comparison to astronauts.- Significant changes in weight and blood pressure after HDT trial, could possible underlie changes in GMV.- No correlation with behavioral tasks investigated.- No control group.
Roberts *et al.* ^69^	8	MRI (1.5 T, volumetric analysis) before and after 90* days of −6° HDBR.	- No changes in GM, WM, CSF or ventricular volumes.- Upward shift and posterior rotation of brain relative to skull.- Correlation between HDBR-induced changes in ventricular volume and posterior brain rotation.- Increase in brain tissue density increases in vertex, including fronto-parietal lobes, with contraction of adjacent extra-axial CSF spaces.- Decrease in brain tissue density in brain areas along the base, including orbitofrontal cortex.- Inter-subject variability.	- Relatively small sample size.- Relatively young age in comparison to astronauts.- Mixed gender study population, possible bias.- Retrospective volumetric brain analysis.- Not clear if correction for multiple comparison (VBM) was applied.- No correlation with behavioral tasks investigated.- No control group.
Cassady *et al.* ^71^	17**	rsfMRI (3 T) before, during and after 70 days of −6° HDBR.	- Increased RSFC during HDBR and decreased RSFC after HDBR between motor and somatosensory brain regions.- Increased RSFC during HDBR between right OP2 region and IL cerebellum.- Decreased RSFC between temporoparietal regions.- Decreased RSFC between cerebellar regions.- Correlation between motor-somatosensory network connectivity and standing balance performance.- Changes in spatial working memory performance.- Changes in RSFC between sensorimotor and SFG, paracingulate gyrus and lateral occipital areas.	- Relatively young age in comparison to astronauts.- HDBR participants and control subjects were not assessed at identical intervals.- Control data was acquired on a different MRI scanner.- Control subjects may have not experienced same emotional reactions associated with HDBR, i.e. decreased sensory stimulation and social interaction.
Yuan *et al.* ^72^	18#	rsfMRI (3 T) before, during and after 70 days of −6° HDBR.	- Increased activation during dual tasking in frontal, parietal, cingulate, temporal and occipital cortices.- Increased activation differences between dual and single task conditions during HDBR relative to before or after HDBR.- Positive correlation between dual-task reaction time and dual-task brain activation in cerebral and cerebellar regions.- Effect of HDBR on brain activation takes place very quickly after onset of HDBR.	- Relatively young age in comparison to astronauts.- HDBR participants and control subjects were not assessed at identical intervals.- Control data was acquired on a different MRI scanner.- Control subjects may have not experienced same emotional reactions associated with HDBR.

# 18 subjects were included in the HDBR study; however, another 12 subjects were enrolled in a control group who underwent a similar neuroimaging and behavioral protocol, but did not experience HDT.

*ACC* anterior cingulate cortex; aINS: anterior insula, *ALFF* amplitude of low-frequency fluctuation, *BART* balloon analog risk task *BL* bilateral, *GMV* gray matter volume, *DC* degree centrality, *fMRI* functional MRI, *HDBR* head-down bed rest, *HDBRx* day x of HDBR trial, *IFG* inferior frontal gyrus, *IL* ipsilateral, *IPL* inferior parietal lobule, *L* left, *MCC* middle cingulate cortex, *MFG*: middle frontal gyrus, *OP2* parietal operculum 2, *PCC* posterior cingulate cortex, *R* right, *ReHo* regional homogeneity, *RSFC* resting-state functional connectivity, *rsfMRI* resting-state functional, *MRI* magnetic resonance imaging, *SFG* superior frontal gyrus, *SMA* supplementary motor areas, *T* tesla, *TBSS* tract-based spatial statistics, *TMS* transcranial magnetic stimulation *VBM* voxel-based morphometry, *VMPFC* ventromedial prefrontal cortex, *WM* white matter.

*4 subjects underwent HDBR for 90 days, while the study had to be stopped earlier for the other subjects who underwent HDBR for 42, 44, 49 and 52 days.

**17 subjects were included in the HDBR study, however, another 14 subjects were enrolled in a control group who underwent a similar neuroimaging and behavioral protocol, but did not experience HDT

### Neuroplasticity and spaceflight

**Fig. 3:**
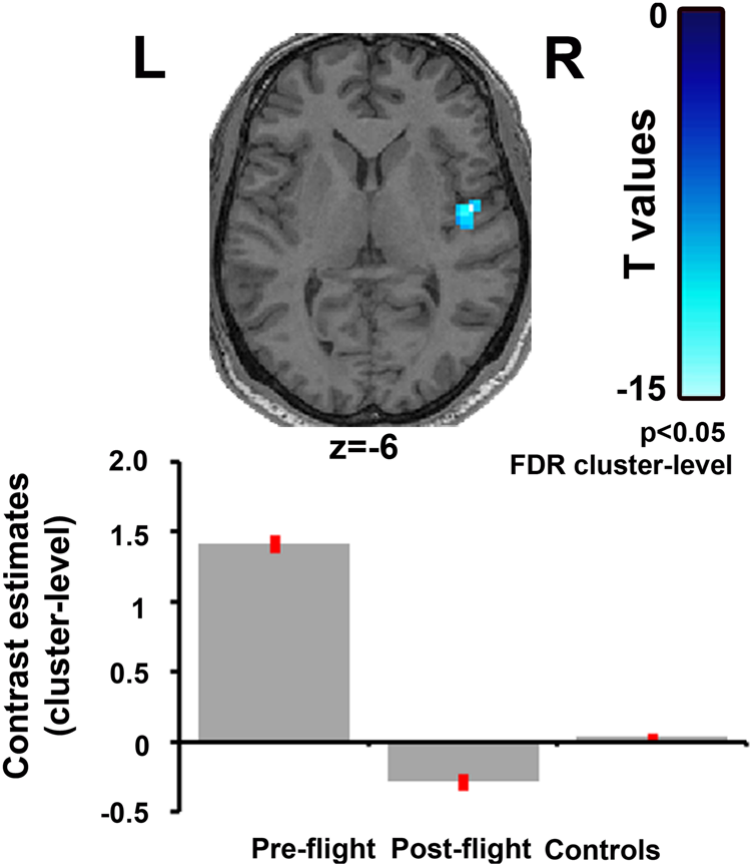
The figure shows decreased connectivity strength in the right insula, a critical region of the vestibular cortex, when comparing post-flight to pre-flight in a cosmonaut. The bars represent the average connectivity strength in the respective cluster with 90% confidence interval (whiskers) for the pre-flight and post-flight scan. The statistical map is rendered on the normalized MRI scan of the cosmonaut (axial view) (from,^60^ used with permission)

So far, there has only been one study examining the neuroplastic effects after actual spaceflight by means of MRI. In this single-subject case study, it was shown, by means of rsfMRI, that long-duration spaceflight is associated with alterations in cerebellar-motor connectivity, as well as a decrease in vestibular connectivity, more specifically a decrease in intrinsic connectivity strength in the right insula (Fig. 3).^60^ This case report showed that the typical spaceflight-related problems such as space motion sickness, postural instability and disorientation could not solely be attributed to the peripheral end organs, i.e. the vestibular system in the inner ear, but may also have a central cortical component. However, interpretation and generalization should be very carefully made due to the anecdotal evidence. On-going longitudinal studies are aiming to extend these preliminary investigations in a larger cohort of astronauts.

### Neuroplasticity and space analogs

Up until now, no MRI-based studies with dry immersion have been performed. In addition, there are no published MRI-based PF studies in humans.

Concerning HDBR, Roberts and colleagues were the first to implement a MRI-based study.^57^ They investigated whether simulated gravity by means of 90 days of HDBR induced changes in functional brain connectivity. In addition, they investigated corticospinal tract excitability by means of TMS. In summary, they found reduced cortical activity in the motor areas with leg representation and a decrease in corticospinal excitability after HDBR. According to the authors, these reductions in cortical motor function could underlie motor-related difficulties in astronauts. Additionally, in the post-HDBR period, they continued TMS and reported an increase in corticospinal excitability. Interestingly, they observed that the larger the increase in motor cortex excitability, the smaller the functional mobility impairment, leading them to assume that TMS could be used as a possible countermeasure against lower extremity dysfunction. Additionally, their findings could be of clinical importance, e.g. pertaining to immobilized patients or patients with lower extremity disuse.

Liao *et al*. initiated a HDBR study in which they investigated short-term alterations in functional connectivity.^56^ After 72 h, they found decreased thalamic connectivity during resting-state, which they attributed to reduced motor control abilities and decrements in executive function in astronauts. In a follow-up study, they corroborated further on their initial results by linking them with a mental transformation test, during which the ability to perform a mental rotation strategy (i.e. mentally rotate an internal representation) is assessed.^61^ Interestingly, they found a correlation between intrinsic connectivity in the left inferior parietal lobe (IPL) and the mental transformation task. In addition, they found a decreased regional homogeneity (ReHo) in the IPL region, known to be involved in mental rotation strategies,^62^ which could explain the decrease in mental function in microgravity. Their study is interesting for the fact that they combined neuroimaging with behavioral data for the first time in regards to (simulated) spaceflight, providing an interesting insight into the link of changes in cognitive function and their underlying neural correlate.

In another fMRI study, Rao and coworkers investigated whether bed rest would influence an individual’s risk-taking behavior and the underlying neural basis of this possible effect.^63^ They implemented the Balloon Analog Risk Task tool^64^ to assess risk-taking. In general, they found no effect of bed rest on risk-taking behavior; however, they did find a significant deactivation of the ventromedial prefrontal cortex (VMPFC) post-HDBR when compared to before. The VMPFC is a principal component of the decision-making circuitry during risky decision-making. The finding of less deactivation of the VMPFC after HDBR is in accordance to the assumed neural adaptation process and changes in neuroplasticity after spaceflight. Furthermore, risk-taking is a high-level cognitive function and therefore, plays an important role in extreme and demanding environments such as spaceflight. Therefore, their results are highly relevant, as they suggest a detrimental effect of (simulated) spaceflight on riskfull decision-making.^63^

Zhou and colleagues performed a study in which they investigated 16 healthy male individuals before and after 45 days −6° HDBR.^65^ They found changes in the anterior insular and middle cingulate cortex (MCC) network, both key regions of the resting state network, that they attributed to the induced cephalic fluid shift and the concurrent increase in CBF, intracranial pressure and oxygenated hemoglobin. In addition, the authors also suggested decreases in autonomic nervous function (i.e. sympathetic and parasympathetic) as another plausible explanation for the underlying decreases in intrinsic functional connectivity in the aINS and the MCC network. Furthermore, they postulated that the decreased anti-correlation with the superior frontal gyrus, a part of the default mode network, together with the decreased correlation within the aINS-MCC network could be the underlying neural correlates of the previously observed alterations in cognitive function occurring during microgravity. Lastly, they did not find any association with emotional state after their 45-day HDBR study. In their study, they presented a very detailed and thorough analysis of the underlying neural correlates in simulated microgravity.^65^ Although they did not include a direct control group as such, they still validated their results by means of an independent data set acquired in healthy male volunteers, not exposed to head-down tilt bed rest, at different time points. However, like all simulated studies, it lacks the direct comparison to actual spaceflight. Spaceflight remains a unique model that even the best simulation model can’t substitute and therefore, all space analog studies most likely underestimate and deviate from the complexity and multi-modal effects of human spaceflight.

Recently, Liao and colleagues published their findings from a rsfMRI study in subjects that underwent a 7-day HDBR experiment.^66^ They postulated that their findings, i.e. reciprocal alterations in the posterior cingulate cortex and anterior cingulate cortex, respectively a decrease and an increase, could account for changes in the autonomic nervous system, as seen in space travelers. In addition, they found an increase in functional activity in the left cerebellar posterior lobule, which could indicate a compensatory role by the cerebellar posterior lobule to overcome the concurrent decline in functional connectivity in the paracentral lobule. This compensatory role of the cerebellum is postulated to be necessary to sustain adequate fine motor control and could be transferred to astronauts in a microgravity condition, where fine motor control is known to be significantly hampered.^67^

In another study, Li and co-workers demonstrated that 30 days of HDBR is associated with local GM and WM alterations.^68^ More specifically, they found decreases in GM volume (GMV) in the bilateral frontal lobes, temporal lobes, parahippocampal gyri, insula and hippocampus, while observing increases in GMV in the vermis, the paracentral lobules, precuneus gyrus, precentral and postcentral gyri. They related these GM changes to the decline seen in performance, locomotion, learning, memory and coordination in space travelers. Their findings should be interpreted cautiously, as their subjects experienced significant changes in weight and blood pressure after the HDBR trial, which could possibly underlie the changes in GMV.

Roberts and colleagues recently published their results from a volumetric MRI analysis in 8 subjects after long-term HDBR.^69^ They found several structural changes due to the simulated microgravity, with the most prominent one being the fact that the brain underwent an upward shift and posterior rotation relative to skull. Furthermore, they found a correlation between the posterior brain rotation and ventricular volume. The authors relate this to a change in CSF homeostasis and urge for further research in order to determine the exact role of this when it comes to the VIIP syndrome and its concurrent symptoms such as increased intracranial pressure, headache and visual impairment. However, a recent review doubts the feasibility of HDBR studies to investigate the effect on vision.^70^ Since there is no loss of tissue weight during HDBR (and any other spaceflight analog for that matter), long-duration HDBR is not a good analog for studies on vision impairment. In addition, no previous HDBR studies have reported vision impairments.^70^ Therefore, HDBR might not be the best model to assess VIIP syndrome and other vision-related impairments and a link with spaceflight should be made cautiously.

Very recently, a 70-day study investigated the effect of long-duration HDBR on brain connectivity and behavior in 17 participants.^71^ A behavioral assessment as well as rsfMRI scans were conducted at 7 time points: two measurements pre-HDBR, three measurements during HDBR and two measurements post-HDBR. In addition, a control group of 14 subjects was added to the study, to take into account the effects of time and practice. Interestingly, this set-up allows investigating not only the changes in brain connectivity after HDBR compared to baseline, but also the temporal changes during the HDBR. The authors reported changes in functional brain connectivity in vestibular, sensorimotor and somatosensory networks. More specifically, they observed connectivity increases during HDBR, followed by decreased connectivity after HDBR, in the motor and somatosensory cortices. The latter might imply a possible adaptive response to the HDBR environment. Therefore, the authors suggest it is plausible that motor control regions play a crucial role in this adaptation to HDBR, which is corroborated by the findings by Roberts and colleagues that 90 days of HDBR are associated with an increased motor cortex activity during foot movement immediately after HDBR and a subsequent reversal of these changes after a recovery period.^57^ In contrast, decreases in brain connectivity were observed between the temporoparietal regions, part of the vestibular network, and an increased functional connectivity between the right parietal operculum 2, a key region of the vestibular cortex,^25^ and the ipsilateral cerebellum. These findings, in conjunction to the earlier described results from^61^ and,^60^ suggest that spaceflight-related sensorimotor problems can be attributed to cortical changes at the central level. Moreover, the previously observed diminished functioning of the peripheral neurosensory organs^22–24^ could also be due to a central inhibition of disturbing erroneous signals coming from the vestibular organs. Furthermore, Cassady and colleagues linked their brain connectivity data with behavioral data and reported a correlation between motor-somatosensory network connectivity and standing balance performance, i.e. an individual with the greatest increase in connectivity strength between the motor and somatosensory cortices demonstrated least behavioral impairment following bed rest. This result, together with the findings from Roberts and colleagues,^57^ suggests that changes in body orientation and unloading, as seen in HDBR, may induce compensatory neural processes,^71^ a finding highly relevant for spaceflight and future space missions. Moreover, it might be the case that individual variability in neural adaptation compensates for the detrimental effects of HDBR, and spaceflight in that matter, more in some participants than in others.^71^

The same research group also investigated the effect of long-duration HDBR on dual task performance and the underlying brain activation.^72^ They found increased brain activation in the frontal, parietal, cingulate and temporal cortices for dual task execution during HDBR, with a recovery to baseline levels after cessation of the HDBR. The latter implies a reduced neural efficiency in this spaceflight analog. This lower neural efficiency has been shown already during spaceflight by means of EEG recordings^73^ and therefore, the HDBR findings seem to be transferable to spaceflight. In addition, the aforementioned study showed that HDBR resulted in nearly immediate changes in brain activation.^72^ Therefore, future studies should also focus on the temporal dynamics of spaceflight-induced neuroplasticity, as indicated by these Earth-based model findings. As discussed above, preliminary spaceflight results have also found a similar effect after 6 months of spaceflight,^60^ but it is unknown if prolonged spaceflight has a linear or exponential effect or after which time the effects level off. A better understanding regarding the temporal characteristics of neuroplasticity is of major importance for future manned missions to the Moon and Mars.

In regards to all above-mentioned studies, it must be mentioned that HDBR induces a cephalic fluid shift that might increase CBF and thus, change the hemodynamics of the brain.^74^ Furthermore, also the increased intracranial pressure and oxygenated hemoglobin might alter brain hemodynamics. Therefore, this alone might already induce changes in the brain and might underlie some of the changes found in the above-mentioned studies. However, one could argue to expect more global changes in structural and functional connectivity due to fluid shifts, rather than regional specific and localized changes as described in the studies above.

Overall, we conclude, at this point of research, the HDBR analog has primarily shown alterations related to motor-related tasks (e.g. fine motor control^66^) and more advanced cognitive function such as executive function,^56^ mental transformation,^61^ spatial working memory^71^ and dual tasking.^72^ Consequently, most studies found changes in sensorimotor, somatosensory and cognitive-related brain regions (for a full overview, see Table 1 and Fig. 2). In addition, a study in actual microgravity have additionally shown the alterations in vestibular-related cortical areas such as the insula.^60^ However, conclusions in regards to spaceflight need to be made carefully by both the indirect comparison of space analogs to actual spaceflight^56,61,63,65,66,68,71,72^ and the small sample size in some of the current studies.^57,60,69^

## General difficulties and limitations of space research

Several HDBR studies found a large inter-subject variability.^57,69^ Previous spaceflight studies have already shown that inter-subject variability in space travelers is quite high, also for other physiological processes such as sensorimotor adaptation^75,76^ and vestibular and otolith deconditioning.^77,78^ High inter-subject variability is therefore a feature that should be kept in mind when analyzing and interpreting spaceflight studies, in particular with regards to studies on spaceflight-induced neuroplasticity. Earth-based studies have already shown that neuroplasticity is a process that is highly individually dependent and is related to several factors such as demographics (e.g. age and gender), genetic variation^35^ and physical activity.^79^

In the same line, it should be taken into account that microgravity effects on brain activation have been shown to be task dependent, as found by previous EEG studies.^37,73^ Therefore, the factors found to be influencing neural activation during simulated spaceflight might not only differ from actual spaceflight, they might also differ per individual and might be dependent on the specific task being executed, e.g. during task fMRI protocols.

Several other limitations are also inherent to space research with the most prominent being the small sample sizes. Up to date, approximately 150 crew members have spent 6 months in the International Space Station (ISS), of which US astronauts [National Aeronautics and Space Administration (NASA)], Russian cosmonauts (ROSCOSMOS) and astronauts from the other space agencies (ESA, Canadian Space Agency, Japan Aerospace Exploration Agency).^80^ Space shuttle missions comprised more crewmembers, but the amount of time in space was not more than 2 weeks, limiting also its effects, and the space shuttle program was suspended in 2011. Unfortunately, it is very difficult to acquire data in a large group of space travelers within a reasonable time frame. Therefore, it takes quite some time for most studies to get up to an acceptable sample size, which can lead to changes in setting, equipment and team members. As an example with regards to MRI-based studies, longitudinal studies could lead to variability in MRI acquisition parameters between scans and therefore, potentially confound observed changes.^81^ In addition, MRI acquisition technology changes rapidly and state-of-the-art pre-processing and statistical analysis techniques develop at a fast rate.^82^ Therefore, a longitudinal study over a long period of time could lead to the fact that out-dated techniques are being used for consistency among measurements.

In addition, also due to logistic restrictions, it is very difficult and often impossible to assess space travelers in the first few hours or days after returning from space due to restrictions in the schedule of astronauts. For neuroplasticity measurements, it could be possible that there is a critical time frame within which changes are detectable by means of MRI measurements. Also, when assessment can only take place a couple of days after returning to Earth, one is not only measuring the spaceflight-induced changes, but also the changes taking place due to re-adaptation back on Earth.^60^ This can hamper the detection of more subtle changes or can even counteract these processes in some cases. Especially in the framework of neuroplasticity, it is known that changes can take place on a very short period of time, e.g. alterations in WM structure can already take place after 90 min of a spatial learning task.^83,84^ Therefore, neuroplasticity assessments must be made at well-considered and repeated time points. This is also relevant for studies in which a spaceflight alternative is implemented, however, in general the logistic and scheduling restrictions are easier to overcome or adjust compared to spaceflight.

When focussing on neuroplasticity measured by MRI only, we can only assess the human brain before and after spaceflight. Due to loads of technical, logistic and payload restrictions, there is no possibility to take an MRI-scanner into space or to the ISS. Therefore, it is not possible to assess neuroplastic events, probed by MRI-techniques, during spaceflight, although this would lead to very interesting insights. However, we could complement before-after MRI assessment with more portable neuroimaging techniques on board such as EEG, TMS or near-infrared spectroscopy (NIRS) and by correlating post-spaceflight changes as measured by MRI with behavioral measurements taken on board.

Another complicating factor is the specific demographic profile of space travelers. In general, there is a well-known majority of male space travelers compared to female space travelers with a ratio of roughly 9 to 1 respectively^85^ (In addition, the mean age of astronauts on their first-time flight to space is slightly different for males and females: 44.5 years vs. 42.5 years^80^). It is therefore important that Earth-based space analogs take this into account in order to resemble the demographic profile as much as possible. It is also known that gender can have an impact on the adaptation of several physiological systems to spaceflight.^80,86–88^ Previous studies on neuroplasticity showed that gender and age could influence the degree and extent of neuroplasticity. The menstrual cycle for example can impact on structural and functional neural adaptations.^89^ Therefore, if space analog studies on neuroplasticity want to transfer their findings to make assumptions or conclusions on spaceflight-induced neuroplasticity, they should match age and gender features as much as possible.

## Implications for countermeasures and neuroimaging in spaceflight-related studies

We should aim to accurately determine and map the effect of changes in brain structure and function on the motor, vestibular and cognitive system in order to make long-duration missions (e.g. during several years) feasible and possible. In a second phase, suitable countermeasures should be determined and applied. The ability to perform landing and post-landing tasks (e.g. on Mars) may be hampered by impaired motor control, movement and motor coordination. This could encumber crew performance, crew safety and may even compromise the mission. Furthermore, higher cognitive tasks (e.g. working memory, risk-taking and dual-tasking) might be influenced, possibly leading to unacceptable risks and hazards in spaceflight, where there is a high working load and stress situations might occur frequently and/or suddenly.

### Countermeasures

Recently, the idea of motor imagery (MI), an experimental paradigm already widely used in sports, has been proposed as an inexpensive and rather simple approach to prepare space travelers for the absence of gravity they will encounter.^90^ MI is a process during which a specific and pre-decided action is internally reproduced in working memory, from a first-person perspective, without any overt motor output.^91^ It typically includes multiple sensory modalities, e.g., mentally visualizing a specific motor task and mentally feeling muscle contractions.^92^ Imagined and executed movements have been shown to have the same vividness and temporal structure^93,94^ and in addition, it has been proven that MI activates similar brain regions as is the case with executed movements, e.g. primary and secondary motor cortices, posterior parietal cortex, basal ganglia and the cerebellum.^95,96^ This kind of mental practice could be applied to prepare astronauts to the sudden absence of gravity and to the re-adaptation phase when coming back to Earth.^90^

Additionally, the study from Roberts and colleagues showed TMS to be a possible countermeasure.^57^ TMS is portable and therefore, possible to be implemented in space. The authors suggest TMS to become part of a countermeasure regime for astronauts on long-duration space missions to counteract lower extremity dysfunction,^57^ e.g. prior to operations on a planetary surface as might be the case for interplanetary missions. Another topic well discussed among space researchers, is artificial gravity as a countermeasure. By introducing continuous or intermittent exposure to artificial gravity (or some sort of gravitational levels), the adaptation to e.g. Martian gravity or re-adaptation to Earth’s gravity might be facilitated.^97^ For example, this could be done by introducing a centrifuge on board.^98^ By doing so, the physiological deconditioning, as seen after exposure to weightlessness, could be counteracted. Undoubtedly, this will also affect the human brain and the underlying neural adaptation to spaceflight. Future studies should investigate to what extent artificial gravity (by means of centrifuge or otherwise) plays a possible role in neuroplasticity.

### Neuroimaging

In regards to neuroimaging specifically, the current literature suffers from the fact that all studies are using different acquisition, data pre-processing and statistical analysis techniques, as well as a different set-up for their experiments. Furthermore, several different analysis techniques such as for example BOLD connectivity measures using hypothesis-driven seed-voxel analyses or data-driven independent component analyses; amplitude of low-frequency fluctuation or ReHo measures have been used, adding to the difficulty to compare different studies with each other. However, since these are analysis happening at the post-processing level, they allow for re-analysis and comparison with more widely used connectivity approaches.

In addition, and this holds true for the majority of neuroimaging techniques: all of the above are indirect measures of synaptic neural activity.^48^ For example, changes in brain volume found with volumetric analyses tools do not allow the possibility to make a conclusion on changes at the cytoarchitectonial level. Moreover, changes in GM (and WM to some extent) could be the result of changes in neuropil, changes in neuronal size, dendritic and axonal adaptations, as well as be related to folding or the development of thicker GM.^99^ In addition to the complexity of the precise origin of GM changes, various factors are known to have an impact on brain morphology and may therefore cause brain volume changes. Also, the difference between short-term and long-term exposure to (simulated) microgravity is of course very relevant, but this intrinsically hampers the comparability among studies. Data sharing and weighted meta-analyses could be proposed for future analyses.

Cognitive changes due to spaceflight might be associated with metabolic changes, even before the occurrence of “clinical” symptoms and this relation should be further examined by means of state-of-the-art techniques such as PET scans^32^ or MRI spectroscopy. These techniques could probe changes in neurotransmitter systems e.g. dopamine receptor activity. Based on findings from earlier animal studies related to spaceflight, it is hypothesized to find changes in humans as well.^30,100^ Earth-based studies have shown that changes in neurotransmitters have major implications for attention,^101^ (long-term) memory,^102^ arousal^103^ and motor activity.^104^ Determining neurotransmitter and hormonal imbalance in space travelers is therefore important to get fundamental insight into how the central neural system adapt to microgravity and in addition, to get insight into the relation between these alterations and behavioral processes.

In relation to spaceflight, it is needed to determine the temporal profile and longevity of neuroplastic changes and correlate these with the temporal profile of the (re-)adaptation process and possible detrimental changes. Therefore, in vivo neuroimaging techniques such as MRI and EEG are crucial as they allow mapping structural, functional and metabolic events in the human brain in relation to microgravity and spaceflight. Gaining insight into the dynamic properties of the human brain over time could also help in the development and application of countermeasures as well as help to determine when or how long they should be applied.^105^

In preparing for (very) long-duration interplanetary missions, it is important to determine the impact of changes in brain structure and function on sensori-motor, higher cognitive and psychological capacities of space travelers, since brain alterations might interfere with the decline in brain volume and functional reorganization and connectivity as seen in a normal ageing population.^106–108^ If this is the case, this might potentially lead to accelerated cerebral aging effects and concurrent accelerated decline, e.g. sensory impairment, motor slowing, memory problems, deficits in attention and processing speed and anxio-depressive disorders (e.g., 112,113).

In general, simultaneous and independent multimodal neuroimaging is pivotal to acquire a still lacking understanding of functional and structural brain processes in relation to human spaceflight. The combination of different complementary electro-physiological and neuroimaging techniques should be used to acquire non-redundant information, e.g. structural, functional and metabolic MRI pre and post spaceflight combined with high-density EEG, TMS and/or NIRS. Not only would this give a more complete insight into spaceflight-induced neuroplasticity, but also would the simultaneous use of different techniques overcome limitations inherent to one single technique. An example of this is combining EEG and MRI for a more efficient assessment of the temporal dynamics and spatial information of the underlying neural processes taking place, i.e. to improve and optimize spatio-temporal resolution.^109^

Another feasible approach would be to validate several motor-related and cognitive tasks on Earth by means of fMRI, which would then allow making predictions on brain alterations when performing these tasks inflight in the ISS for example. Illustrations are tests for sensorimotor skills, attention, working memory, spatial orientation, etc. These can be easily done on board of the ISS since they are portable, non-expensive and non-time consuming. A good example hereof is the “Cognition” test battery that is currently being implemented by NASA.^110^

## Conclusion and future perspectives

In conclusion, despite the discussed limitations of the current literature regarding heterogeneous experimental set-ups and the lack of comparability of findings among studies, some trends have been witnessed. The cerebellum, cortical motor areas and vestibular-related pathways seem to be critically involved across different studies, indicating that these brain regions are indeed affected by real and simulated spaceflight. These changes reflect most likely an underlying neural component of the common detrimental changes observed in space travelers such as problems with sensorimotor control and motor coordination, space motion sickness and a hampered otolith and vestibulo-autonomic functioning.

Currently, there is paucity in the knowledge of the effect of microgravity on the human brain and more extensive research is therefore highly needed to increase and add more insight into this matter. The relationship between spaceflight-related physiological and neuro-psychological problems and alterations in brain structure or function should be investigated. Elaborating on the understanding of how the brain reacts to and behaves in spaceflight is a crucial step in the development of more adequate countermeasures against the detrimental changes often seen in space travelers. Assessing space travelers by means of validated and standardized multimodal neuroimaging protocols will help establish a more precise picture of functional, structural and biochemical brain alterations associated with spaceflight. Hereto, it could be of interest to develop a protocol comprising of the minimum of tests that should be performed to optimize merging among studies as much as possible. Within the framework of the space agencies, an international multi-disciplinary task-force or topical team should be established to set-up such a list.

Extending this knowledge is pivotal to guarantee the safety and efficiency of future space missions, such as interplanetary missions to Mars and the development of permanent space habitats. Furthermore, the development, safety and success of commercial space tourism are dependent on how a less-trained human being reacts to this short-term exposure to microgravity, including possible alterations at the level of the brain. Lastly, the acquired insights in this unique population of space travelers have direct and indirect clinical impacts and could be transferred to multiple neurological and psychiatric diseases and pathologies on Earth such as patients suffering from neurodegenerative disorders, vestibular problems and motor immobilization.
